# The Tenure of Sanitary Office

**Published:** 1905-02-25

**Authors:** 


### The Tenure of Sanitary Office.

In what may be described as the earlier period of modern sanitary legislation, dating from the passage of the Public Health Act of 1872, a large amount of activity was directed towards defeating the intentions of the Legislature. The head of the department of State to which the administration of the new law was entrusted was commonly believed to have been selected with especial reference to his inclination to render its provisions nugatory, while, at the same time, the owners of nuisances spared no pains to secure the election of themselves and their friends as members of the various local authorities upon which duties relating to the preservation of the public health were imposed, and by which, in consequence, they were in many places systematically ignored or neglected. A weapon of great importance was placed in the hands of the various boards so constituted by those provisions of the law which rendered the sanitary officials of these boards removable at pleasure by the bodies appointing them. A sanitary officer, in such circumstances, was expected to be seen and not heard, and any tendency to elevate his voice was almost certain to be followed by speedy dismissal. In the course of thirty years, there has, no doubt, been a considerable enlightenment of the public mind concerning the value of sanitary precautions and services; and an amount of zeal and activity is now expected from officers which, if it had been displayed thirty years ago, would have been fatal to their prospects. We are disposed to think, however, that enlightenment has not yet been carried sufficiently far to afford them adequate protection in the discharge of their duties.

Sir Lauder Brunton, in an address which he delivered last month to the Society of Medical Officers of Health, rightly pointed out that the medical officer could not be a trustworthy inspector unless he had reasonable security of tenure, and that, in many small districts, this primary condition of complete efficiency is not at present realised. The same position was taken up, still more recently, by Mr. Young, chief sanitary inspector of the Borough of Battersea, who, in a speech made at the annual dinner of the Association of Sanitary Inspectors, described his negotiations with the executive of the British Medical Association on the subject of a Bill which the Association intends to promote in Parliament, and under which it was thought that security of tenure might be afforded to Sanitary Inspectors as well as to Medical Officers of Health. Mr. Young said that, after the meeting of the Sanitary Inspectors' Association at Bournemouth last autumn, he had been approached by members of Parliament, who asked him for statistics of health officers who had been discharged from their positions for doing their duty. Some cases could, he said, have been supplied, but the real answer to the question was that the fear of the officers lest they should lose their appointments hindered them in the discharge of their duties, with the effect that public health administration was jeopardised. It was, continued Mr. Young, a question of bread and butter, and officers were tempted to rub along easily, just doing enough to please the members of the authority. If this be a correct picture of the present state of affairs the interests of the public are still being sacrificed to those of the owners or creators of nuisances. It is, of course, necessary that the public should be protected against undue officialism; but no one who is acquainted with the circumstances of many localities can question the still more pressing necessity of their being protected against well-known and flagrant causes of disease. Every annual report of the Medical Officer of the Local Government Board contains narratives showing how local authorities, in spite of repeated warnings, have maintained these causes in full operation; and it seems probable that the report of the coming year will contain narratives of this sort in relation to Lincoln and to Cambridge. The position which has been attained by medical officers under the Poor Law, who cannot be dismissed by Boards of Guardians without the consent of the Local Government Board, would be sufficient to meet the case of sanitary officers, and we cannot but think that Mr. Long, whose tenure of the Presidential office has enabled him to afford so many proofs of exceptional enlightenment, would be ready and willing to promote the simple change in the law by which this degree of protection would be afforded. Sanitary officers would, we think, be ill-advised if they were to tack their case on to that of the British Medical Association. The Bill to be promoted by that Association has not yet, we believe, received its final form, although it has been relieved of some of the absurdities by which the original draft stood self-condemned. But it manifestly bristles with difficulties and points of contention, and the past history of medical legislation in this country lends but slender support to the hope that these difficulties will be speedily overcome. Security of tenure could be given to sanitary officers by a Bill containing only a single clause, and there can be little doubt that such a Bill, if promoted by Government, would be at once accepted by Parliament.

